# A Viable Lyopreserved Amniotic Membrane Modulates Diabetic Wound Microenvironment and Accelerates Wound Closure

**DOI:** 10.1089/wound.2018.0931

**Published:** 2019-07-25

**Authors:** Sandeep Dhall, Tyler Hoffman, Malathi Sathyamoorthy, Anne Lerch, Vimal Jacob, Matthew Moorman, Jin-Qiang Kuang, Alla Danilkovitch

**Affiliations:** Research and Development, Osiris Therapeutics, Inc., Columbia, Maryland.

**Keywords:** amnion, lyopreservation, wound healing, inflammation, re-epithelialization, oxidative stress

## Abstract

**Objective:** Wound healing is a complex process involving the dynamic interplay of various types of cells and bioactive factors. Impaired wound healing is characterized by a loss in synchronization of the process, resulting in non-healing chronic wounds. Human amniotic membrane (AM) has been shown to be effective in the management of chronic wounds. Recently, a viable lyopreserved AM (VLAM) has been developed. The VLAM retains the structural, molecular, and functional properties of fresh AM with the advantage of a long shelf life for living tissue at ambient temperatures. The objective of this study was to evaluate the effects of VLAM on the impaired wound microenvironment and wound closure in db/db mice.

**Approach:** VLAM or saline gel (control) was applied weekly to 7-mm excisional wounds in diabetic (db/db) mice. Wound appearance and size were assessed weekly. Inflammation and redox state in wounds were tested by cytokine gene and protein expression, and by catalase and glutathione peroxidase activities, respectively. Wound tissue granulation and neovascularization were assessed histologically.

**Results:** Diabetic wounds treated with VLAM closed faster than control wounds. On an average, VLAM-treated wounds closed 4 days faster than the control wounds, with a significantly faster rate of closure at days 7 and 14 as compared with control wounds. The faster closure correlated with a decrease in the expression of proinflammatory factors and oxidative stress, and an increase in angiogenesis and dermal thickness.

**Innovation:** Effects of VLAM on a chronic wound microenvironment and underlying molecular mechanisms were investigated for the first time.

**Conclusion:** VLAM accelerates wound closure in db/db mice by decreasing inflammation and oxidative stress and supporting wound tissue granulation, neovascularization, and re-epithelialization.

**Figure:**
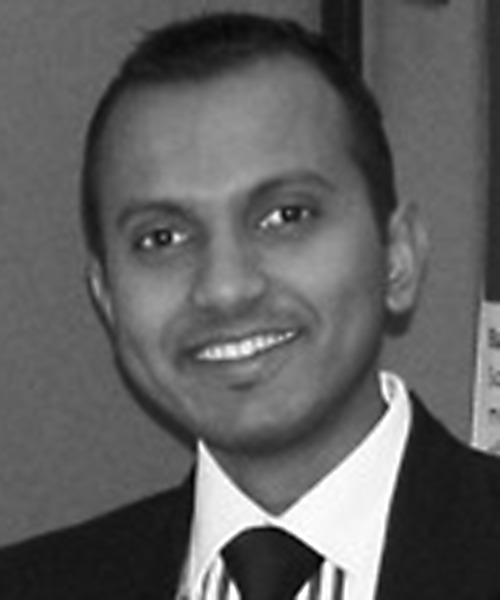
**Sandeep Dhall, PhD**

## Introduction

Normal wound healing is a physiological response to tissue injury. Wound healing is a dynamic, complex process with four overlapping phases: homeostatic, inflammatory, proliferative, and remodeling.^1^ Each phase of wound healing involves coordinated activities of numerous types of cells and bioactive factors. Aging, obesity, smoking, and many diseases, including diabetes, cardiovascular and kidney diseases, have negative effects on wound healing. Impaired wound healing is characterized by a loss in synchronization of the process, resulting in chronic non-healing wounds. Hallmarks of chronic wounds include inflammation, exacerbated levels of proteases and reactive oxygen species (ROS), and biofilm-forming bacteria.^1–4^ The number of people with chronic wounds is rapidly growing, reaching almost 7 million with an approximate annual cost of $25B in the United States alone.^3^ Despite having a broad variety of advanced wound therapies available, chronic wounds remain a treatment challenge.

Human amniotic membrane (AM) has extensive history in chronic wound management. The development of tissue preservation methods has facilitated solving the problem of short shelf life, a major limitation of using fresh AM. At the present time, a number of different AM grafts are commercially available, including viable cryopreserved AM (VCAM), a biomaterial shown to retain the matrix, growth factors, and viable cells in the native state.^5^ Accumulated clinical evidence demonstrates the benefit of VCAM as an adjunct to standard of care in the treatment of chronic wounds, including diabetic foot ulcers.^6^ However, cryopreserved products require the use of ultra-low temperature freezers and shippers for both storage and distribution, limiting widespread use of these products. To address these limitations, we recently developed a lyophilization method for the preservation of living tissues, including AM, to allow for storage at ambient temperatures.^7^ We previously showed that, similar to VCAM, viable lyopreserved AM (VLAM) retains both structural and functional properties of fresh AM.^7^

We hypothesized that VLAM could mitigate inflammation in an impaired diabetic wound environment and lead to proper wound healing. To address our hypothesis, we investigated the effects of VLAM treatment *in vivo* on the wound microenvironment and healing in diabetic (db/db) mice. Using an excisional wound model, we demonstrate that VLAM treatment results in rapid wound closure in these animals. At the molecular level, we find that VLAM reduces inflammation and oxidative stress, resulting in an improved wound microenvironment, faster wound closure, and a better quality of regenerated tissue.

## Clinical Problem Addressed

Cryopreservation is currently the only method for long-term storage of living cells and tissues. Although VCAM has clinical benefits in wound treatment, the requirement of “cold chain” storage and distribution significantly limit its widespread use. We have developed a lyopreservation technique that is reproducible, scalable, and allows for long-term ambient storage of living tissue. Here, we confirm that all the components of fresh AM are preserved in VLAM. We also identify the underlying mechanisms by which VLAM modulates the impaired diabetic wound environment, resulting in rapid wound closure *in vivo*. In conclusion, VLAM is an alternative to VCAM for addressing difficult-to-treat wounds without the constraints of cold chain storage.

## Materials and Methods

### Tissue procurement and ethics statement

Human full-term placentas, commercially available, were provided by The National Disease Research Interchange (NDRI, Philadelphia, PA) and Cord Blood America, Inc. (CBA, Las Vegas, NV). NDRI and CBA provided the tissue procurement and ethics statement. Placentas were collected after obtaining written, informed consent from eligible donors. Placentas for this study were acquired and processed within 2 days.

### Tissue processing and lyopreservation

#### Amnion (AM) isolation

Human normal full-term placentas were processed as previously described.^8^ Briefly, AM was separated from the chorion and umbilical cord, cleaned, and treated with an antibiotic cocktail. Post-residual antibiotic removal using Dulbecco's Modified Eagle Medium (GE Healthcare, Logan, UT), the AM was cut into 25 cm^2^ pieces. VLAM was prepared as previously described by using the lyopreservation protocols described later.^7^ Devitalized lyopreserved AM (DLAM) was prepared by subjecting AM to five cycles of rapid freeze-thawing before processing for lyopreservation.

#### AM lyopreservation

Tissue lyophilization was carried out as previously described.^7^ Briefly, tissue samples were soaked in a 0.25 M trehalose (Avantor, Center Valley, PA) solution at RT for 30 min. Tissue samples were packaged in Tyvek pouches (Rollprint, Addison, IL) and placed in a lyophilizer. Tissue freezing was performed by cooling shelves to −50°C at 1.16°C/min for 120 min. Primary drying was done at 200 millitorr (mTorr) at −20°C for 360 min. A three-phase secondary drying followed the primary drying. The first phase was at 200 mTorr at 0°C for 360 min, the second phase was at 200 mTorr at 20°C for 360 min, and the third phase was at 200 mTorr at 30°C for 2,760 min.

### VLAM and DLAM rehydration procedure

VLAM and DLAM were rehydrated by immersing VLAM and DLAM in sterile Dulbecco's phosphate-buffered saline (DPBS) for 30 min at RT.

### Assessment of VLAM and DLAM cell viability

Fresh AM, and VLAM and DLAM post-rehydration were stained with SYTO 24 and Ethidium Homodimer-1 (Thermo Fisher Scientific, Waltham, MA). A working LIVE/DEAD^®^ nuclear stain was prepared by adding 1 μL of reconstituted SYTO 24 solution and 3 μL of reconstituted Ethidium Homodimer-1 solution per 1 mL DPBS. Sixteen-millimeter-diameter disks were cut from 25 cm^2^ samples of fresh AM, VLAM, and DLAM and they were used for staining. Samples were completely submerged in the staining solution for 5 min. They were rinsed in DPBS to remove excess staining, and cell viability was evaluated by using Invitrogen™ EVOS™ FL Auto Imaging System System and Celleste™ Image Analysis Software (Thermo Fisher Scientific).

### *In vivo* testing

#### Animals

Diabetic (db/db) male mice (Jackson Laboratories, Bar Harbor, ME) were used in this study. Six-month-old animals were housed at the Sobran BioScience vivarium at Johns Hopkins University. Experimental protocols were approved by the Sobran's Institutional Animal Care and Use Committee. All procedures were performed in accordance with the guidelines and regulations of The Association for Assessment and Accreditation of Laboratory Animal Care International. All mice were fed a standard chow diet.

#### Dermal excision db/db wound model

Seven-millimeter-wounds were created as previously described.^9^ The wounds were treated weekly topically with VLAM or DLAM and covered with Tegaderm™ (3M, St. Paul, MN) to prevent wound contamination. Control wounds were treated with saline normlgel^®^ (Molnlycke Healthcare, Gothenburg, Sweden) and Tegaderm (3M). Wound photographs were taken weekly before VLAM and DLAM re-application. The wound area was quantified by using ImageJ (NIH, Bethesda, MD). Tissue samples were collected at various time points after wounding by using a 10-mm-diameter biopsy punch. The collected samples were either fixed in 4% paraformaldehyde and processed for histological evaluation or flash frozen and stored at −80°C for biochemical, cytokine, and RNA analysis.

### Histological and fluorescent immunohistochemical staining procedures

Collected wound tissue samples were fixed in 4% paraformaldehyde. Tissue sectioning and staining were performed by Histoserv, Inc. (Germantown, MD) using standard protocols for hematoxylin and eosin and Masson's trichrome staining. For detection of alpha smooth muscle actin (αSMA), rabbit polyclonal antibodies (#5694; Abcam, Cambridge, MA) were used followed by goat anti-rabbit AlexaFlour 568-labeled IgG (#A-11011; Thermo Fisher Scientific). For detection of cluster of differentiation 31 (CD31), a rat monoclonal antibody (#7388; Abcam) was used followed by goat anti-rat AlexaFlour 568-labeled IgG (#A-11077; Thermo Fisher Scientific). For detection of Collagen Type IV, Collagen IV rabbit polyclonal antibodies followed by goat anti-rabbit AlexaFlour 568-labeled IgG (#A-11011; Thermo Fisher Scientific) were used. Sections stained with goat anti-rat AlexaFlour 568-labeled IgG (#A-11077; Thermo Fisher Scientific) or goat anti-rabbit AlexaFlour 568-labeled IgG (#A-11011; Thermo Fisher Scientific) were used as negative controls. Vectashield^®^ (Vector Laboratories, Burlingame, CA) mounting medium containing 4′,6-diamidino-2-phenylindole (DAPI) was used to mount the slides. DAPI stained the cell nuclei. Both wound closure and histological analyses were performed by investigators blinded to treatment. Quantification of the CD31^+^-stained areas was performed by using ImageJ software. Results were normalized to the total number of DAPI-stained cells present in each tissue section.

### PCR array gene expression and confirmatory PCR

Flash-frozen wound tissue collected at day 7 post-injury was used for PCR array gene expression and confirmatory PCR expression experiments. For gene expression, results were expressed as fold change in VLAM-treated tissues compared with the control group. The Qiagen data analysis web portal was used to calculate fold change/regulation using the delta-delta C_T_ (cycle threshold) method. Delta C_T_ was calculated between the gene of interest and the average of reference genes, followed by delta-delta C_T_ calculations (delta C_T_ (Test Group)-delta C_T_ (Control Group)). Calculation of fold change was done by implementing the 2^ (-delta delta C_T_) formula. For confirmatory PCR expression, isolated RNA was transcribed by using a cDNA conversion kit (Qiagen, Inc., Germantown, MD). The cDNA in combination with RT^2^ SYBR^®^ Green quantitative PCR (qPCR) Mastermix (Qiagen, Inc.) was used with RT^2^ qPCR Assays (Qiagen, Inc.). Gene fold regulation was calculated by using the delta-delta C_T_ method. Fold change was calculated by using the formula 2^ (-delta delta C_T_). GAPDH was used as a reference gene.

### Cytokine/chemokine multiplex analysis of wound samples

Samples of normal skin and wound tissue at days 7 and 14 post injury were collected, and extracts were prepared as previously described.^10^ Tissues in T-PER (Thermo Fisher Scientific) supplemented with a protease inhibitor cocktail (Sigma-Aldrich, St. Louis, MO) were lysed in TissueLyser LT (Qiagen, Inc., Valencia, CA) at 50 oscillations/s by using zirconium oxide beads. Clarified supernatants, obtained post-centrifugation at 14,000 rpm for 15 min, were analyzed for the presence of selected cytokines and chemokines by using a magnetic Luminex assay kit (#LXSAMSM-22; R&D Systems, Minneapolis, MN) whereas Bio-Plex MAGPIX™ (Bio-Rad, Hercules, CA) was used to run the multiplex. The concentration of each analyte was normalized to the total protein concentration of the sample.

### Measurement of antioxidant activity of tissue catalase and glutathione peroxidase in wound samples

Catalase and glutathione peroxidase activities in wound tissue sample lysates prepared at day 7 were measured by using commercially available kits (#707002 and #703102; Cayman Chemical, Ann Arbor, MI) according to the manufacturer's instructions. Results of enzymatic activity were expressed in nmol/min/mL.

### Statistical analysis

Results are presented as mean ± standard deviation. Gene expression is presented as fold change in VLAM-treated tissues compared with the control group. Student's *t*-test was used to determine the significance of differences between groups, whereby *p* < 0.05 was considered significant.

## Results

### VLAM accelerates wound closure in diabetic mice

**Figure 1.**
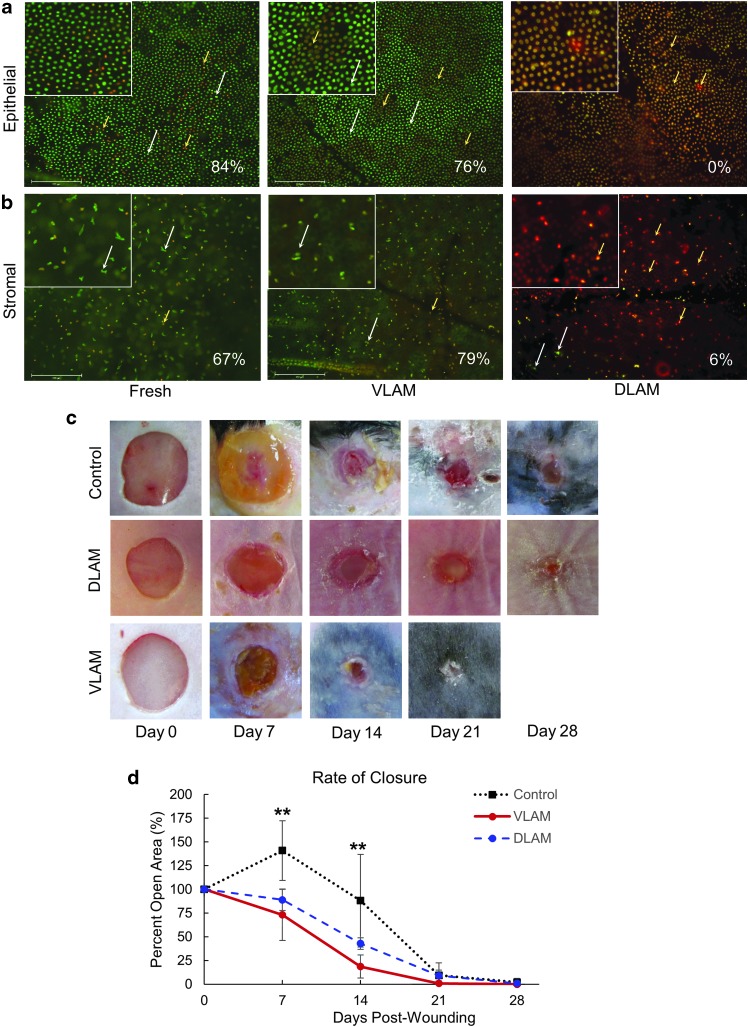
VLAM accelerates wound closure in diabetic mice. The presence of viable cells in VLAM and fresh AM was confirmed by LIVE/DEAD staining. DLAM staining showed that no viable cells were present in the tissue. Viable cells (*green* only, *white arrows*) and dead cells (*reddish*-*yellow* and *red*, *yellow arrows*) in epithelial **(a)** and stromal **(b)** layers. Cell viability (%) is shown for each individual image. Higher magnification *insets* of viable and dead cells in the stained tissue. Representative photographs of wounds taken weekly over a 21-day period post-wounding in control (saline gel), DLAM- and VLAM-treated groups **(c)**. Wound areas were measured weekly and expressed as a percent of the wound area at day 0. Mean ± SD for six mice per treatment group are shown for each time point on the graphs. **p < 0.01 VLAM versus control **(d)**. AM, amniotic membrane; DLAM, devitalized lyopreserved AM; SD, standard deviation; VLAM, viable lyopreserved AM.

A diabetic mouse wound represents the most commonly used model of impaired wound healing: Wounds will close, whereas the rate of wound closure in diabetic mice is significantly delayed in comparison to non-diabetic animals.^11^ We used this model to investigate the rate of wound closure in VLAM-treated animals and compared these data with those in control and DLAM-treated animals. Before using VLAM in *in vivo* experiments, we used the LIVE/DEAD viability/cytotoxicity assay to confirm that viable cells were present in both epithelial and stromal layers of VLAM. The majority of cells in both epithelial (Fig. 1a, middle panel) and stromal (Fig. 1b, middle panel) layers of VLAM remained viable after rehydration. On the contrary, DLAM lacked viable cells in either layer (Fig. 1a, b, right panels). The percentage of viable cells was calculated from LIVE/DEAD microscopic images and expressed per field as: [number of viable cells/number of total cells] × 100%. Fresh AM served as a positive control (Fig. 1a, b left panels). The white arrows highlight the viable cells (green only). The yellow arrows illustrate the red and reddish-yellow dead cells. Overall, there were no significant differences in the percentage of viable cells in VLAM and fresh AM.

**Figure 2.**
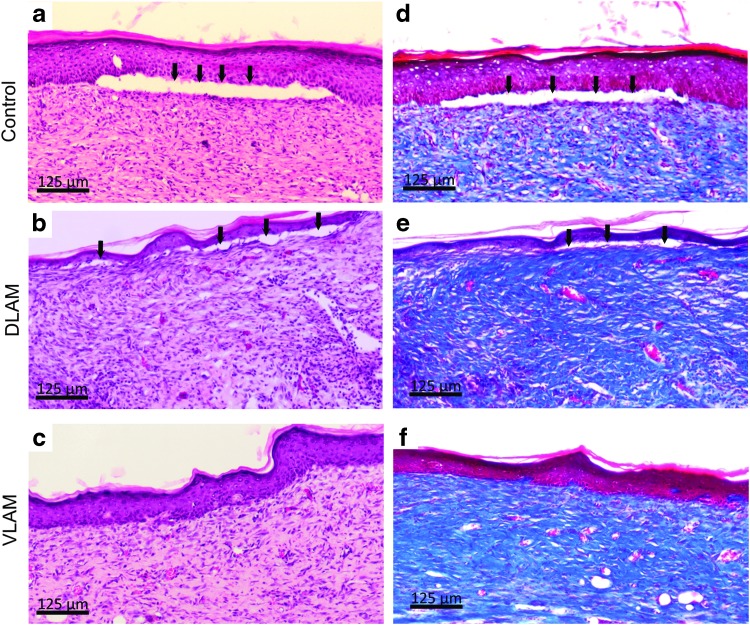
VLAM regenerates skin tissue in a diabetic mouse wound model. The integrity of skin tissue post-wound closure in control, DLAM- and VLAM-treated animals was assessed histologically. Wound tissue post-closure was collected and stained with H&E **(a–c)** or MT **(d–f)**. Skin structures in the control and DLAM-treated wound lack dermal-epidermal junctions **(c,** *black arrows***)**. Complete regeneration of skin tissue was observed in a VLAM-treated wound **(b, d)**. H&E, hematoxylin and eosin; MT, Masson's trichrome.

Figure 1c, d demonstrates that wounds treated with VLAM closed faster than control and DLAM-treated wounds. The rate of wound closure for DLAM was not different from the rate of wound closure observed in control animals (Fig. 1c, upper and middle panels). The rate of VLAM-treated wound closure was significantly faster at days 7 and 14 as compared with control wounds. Therefore, in all subsequent experiments, we compared VLAM-treated wounds with control wounds. In addition to the slower rate of wound closure observed in both control and DLAM-treated animals (Fig. 1c, d), histological analysis demonstrated that after wound closure, a continuous dermal-epidermal junction was not observed (Fig. 2a, b, d, and e). The black arrows illustrate the break in the dermal-epidermal junction. In contrast, a fully restored dermal-epidermal junction was observed in VLAM-treated wounds (Fig. 2c, f).

### VLAM downregulates inflammation and oxidative stress in diabetic mouse wounds

Wounds in diabetic mice are characterized by high levels of inflammation and oxidative stress that prevent wounds from healing.^12,13^ Our findings using VLAM (Fig. 1c) suggest that accelerated wound closure might be due to a modulation in the wound microenvironment. To investigate the effects of VLAM on a diabetic wound microenvironment, we analyzed wound tissue samples collected from VLAM-treated animals and compared those with control wound tissue samples.

**Figure 3.**
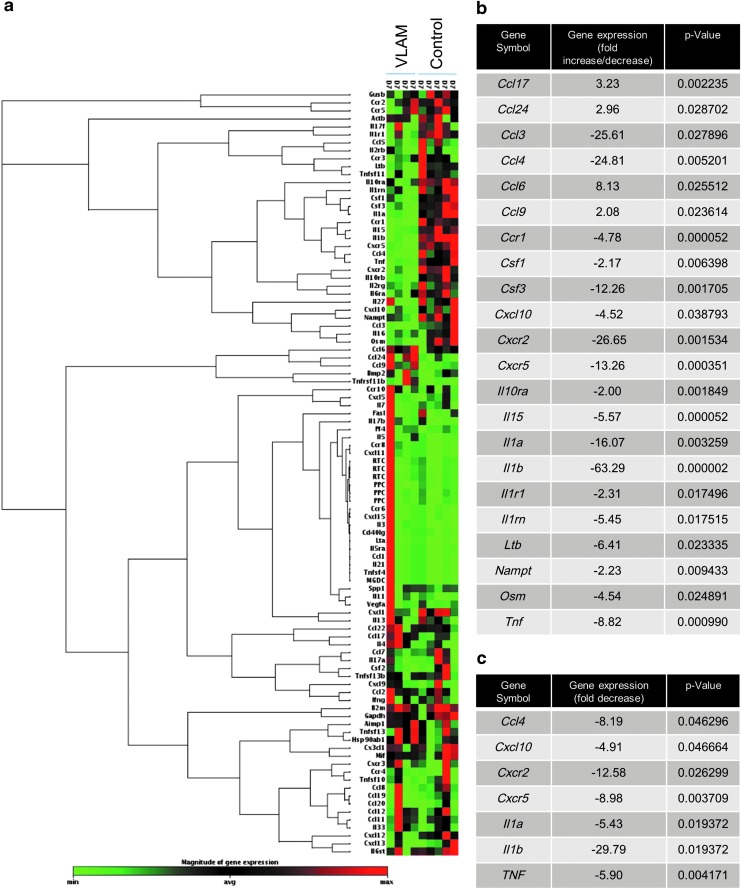
VLAM treatment decreases the expression of proinflammatory genes in diabetic wounds. Expression of 96 mouse wound healing-associated genes at day 7 post injury in VLAM-treated wound tissue (*n* = 4) was compared with that in control animals (saline gel, *n* = 5) **(a)**. Data are presented as a non-supervised hierarchical clustering of genes with a heat map. Individual mouse wound-healing proinflammatory genes with the most significant positive or negative change are shown for VLAM-treated wounds and control wounds **(b)**. qPCR results for expression of seven proinflammatory genes in VLAM-treated wounds are depicted as a fold change compared with saline gel control wounds (*n* = 3 per group). Each assay for each sample was run with four replicates. Average SD was 0.25 **(c)**. qPCR, quantitative PCR.

The cytokine/chemokine profile in wound tissue was assessed by gene expression and PCR and by multiplex protein arrays. Samples were collected on day 7, after one application of VLAM, for the gene expression array and for PCR. For the multiplex protein array, samples were taken on days 7 and 14, after one or two applications of VLAM, respectively. The gene array included 96 mouse wound healing-associated genes. Figure 3a illustrates a non-supervised hierarchical clustering of genes with a heat map visualizing the fold change in genes expressed in VLAM-treated wounds and in control wounds. The heat map shows an increased expression of proinflammatory genes in control wounds (red-colored sections clustered predominantly in the upper part of the clustergram). In contrast, after VLAM application, the expression of genes encoding proinflammatory factors was downregulated: The red-colored section in the upper part of the clustergram in control samples was green in VLAM-treated wounds (Fig. 3a). Individual genes with the most significant positive or negative change in expression after VLAM treatment are listed in the table (Fig. 3b). VLAM downregulates the expression of genes encoding proinflammatory cytokines and their receptors, including tumor necrosis factor-α (*TNF*-α), interleukin-1 alpha (*IL-1*α), interleukin-1 beta (*IL-1*β), and Chemokine (C-C motif) ligand *(Ccl)-3* and -*4*, also known as macrophage inflammatory protein 1-alpha and beta (*MIP-1*α and *MIP-1*β), respectively, C-X-C motif chemokine receptor *(CXCR)-2* and -*5* (Fig. 3b). Downregulation of these proinflammatory genes was confirmed by PCR (Fig. 3c).

**Figure 4.**
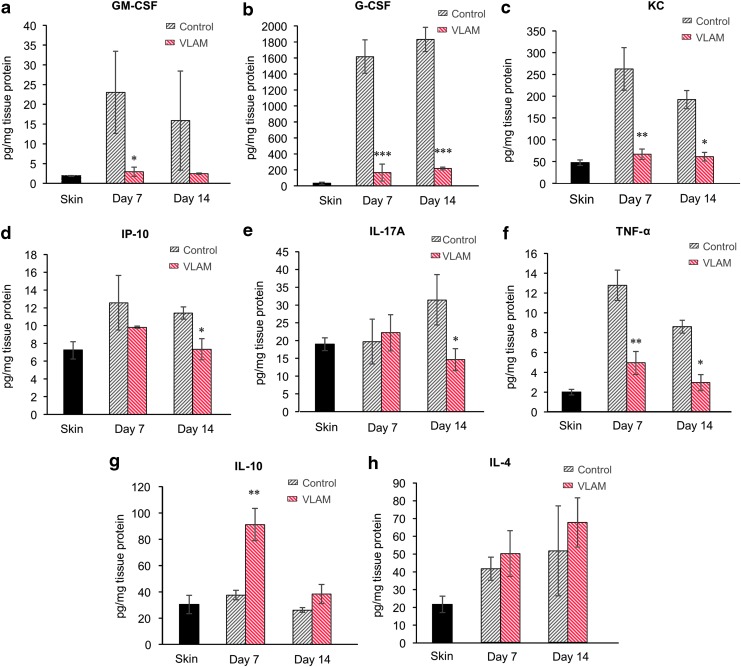
VLAM downregulates proinflammatory and upregulates anti-inflammatory chemokines and cytokines in diabetic wounds. Levels of cytokines and chemokines at days 7 and 14 post-injury in VLAM-treated wound tissue (*n* = 4 per each time point) were compared with control animals (Saline gel, *n* = 4 per each time point) **(a–h)**. Unwounded skin samples (labeled “skin”) served as a reference control for levels of cytokines in normal skin. The concentration of each analyte was normalized to the total protein concentration of the sample. Bar graphs depict mean ± SD. *p < 0.05, **p < 0.01, and ***p < 0.001 compared with control.

A multiplex protein array was performed to evaluate the effects of VLAM on cytokine and chemokine levels in tissues collected from wounds at days 7 and 14. Unwounded skin samples served as a reference control. This analysis included proinflammatory factors granulocyte-macrophage colony-stimulating factor (GM-CSF), granulocyte colony-stimulating factor (G-CSF), keratinocyte chemoattractant (KC), interferon gamma inducible protein 10, interleukin-17A, and TNF-α (Fig. 4a–f), and anti-inflammatory cytokines IL-4 and IL-10 (Fig. 4g, h). VLAM decreases proinflammatory factors (Fig. 4a–f) and increases the levels of anti-inflammatory cytokines (Fig. 4g, h) at both time points (7 and 14 days after application of VLAM). In particular, significant decreases ranging from 2.5- to 160-fold were detected for GM-CSF (Fig. 4a), G-CSF (Fig. 4b), KC (Fig. 4c), and TNF-α (Fig. 4f). In addition, VLAM application resulted in a greater than twofold increase in anti-inflammatory cytokine IL-10 at day 7 (Fig. 4g), and a modest increase in anti-inflammatory cytokine IL-4 at both time points (Fig. 4h).

**Figure 5.**
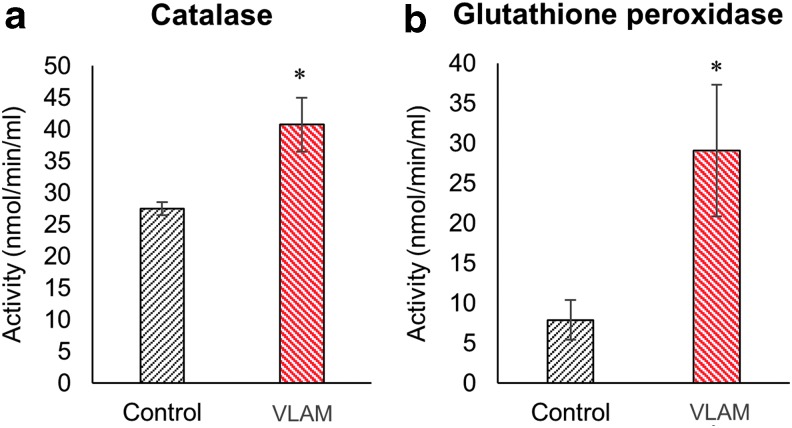
VLAM increases antioxidant activity of catalase and glutathione peroxidase in diabetic wounds. Antioxidant enzymatic activities of catalase **(a)** and glutathione peroxidase **(b)** were measured in control (saline gel) and VLAM-treated wound tissue samples collected at day 7 post-treatment (*n* = 4). Bar graphs depict mean ± SD. *p < 0.05 compared with control.

It is known that chronic wounds have increased levels of ROS, in part, due to a reduction in antioxidant enzymes that scavenge ROS. Similar to diabetic patients with chronic wounds,^14^ wounds in db/db mice are characterized by a high level of oxidative stress and reduced activity of antioxidant enzymes.^9,13^ To determine the effect of VLAM treatment on oxidative stress in diabetic mouse wounds, we examined the activity of antioxidant enzymes catalase and glutathione peroxidase in wound samples collected on day 7. As shown in Figure 5, both catalase (Fig. 5a) and glutathione peroxidase (Fig. 5b) activities were significantly increased in VLAM-treated wounds compared with control wounds.

### VLAM promotes neovascularization and basement membrane formation in diabetic mouse wounds

**Figure 6.**
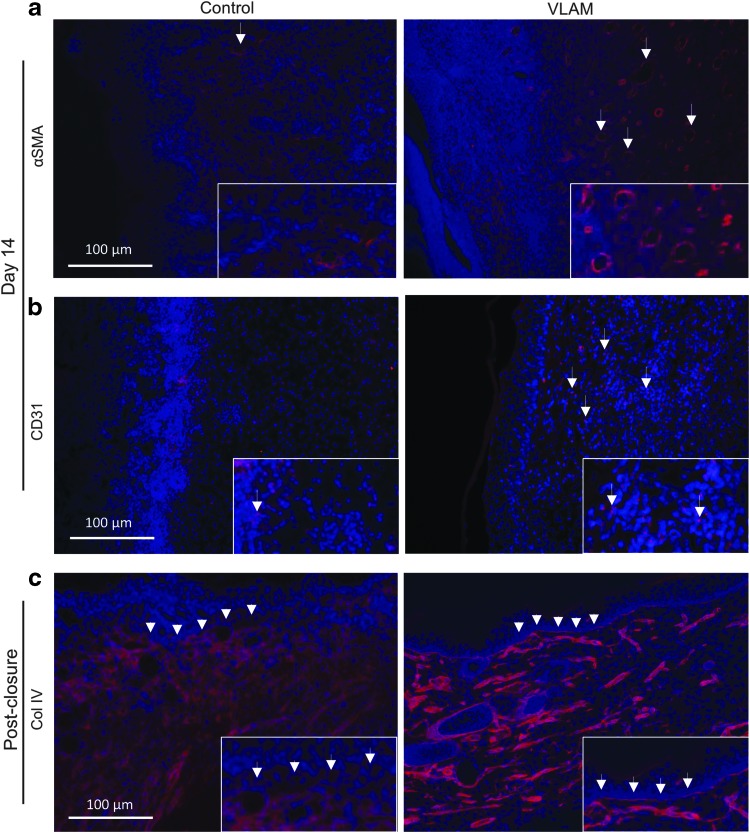
VLAM stimulates new blood vessel and basement membrane formation in diabetic wounds. Wound neovascularization in control (saline gel) and VLAM-treated wound tissue samples collected at day 14 was determined by αSMA **(a)** and CD31 **(b)** staining. *White arrows* (*right top* and *middle panels*) highlight the presence of endothelial cells indicative of new blood vessel formation in a VLAM-treated wound. The basement membrane in control (saline gel) and VLAM-treated wound tissue samples collected post-closure was visualized by collagen IV (Col IV) staining. Higher magnification *insets* of stained tissue sections. *White arrowheads* (*bottom panels*) highlight the basement membrane **(c)**. A total of four animals was used for each group, and tissues were co-stained with DAPI (*blue*) to visualize cell nuclei. αSMA, alpha smooth muscle actin; DAPI, 4′,6-diamidino-2-phenylindole.

To examine whether VLAM supports vascularization, we evaluated wound samples collected at day 14 for the presence of blood vessel cell markers. Samples were stained with fluorescently labeled antibodies for smooth muscle cell marker, αSMA (Fig. 6a), and endothelial cell marker, CD31 (Fig. 6b). DAPI (blue staining) was used to visualize cell nuclei. As shown in Fig. 6, both αSMA and CD31 were markedly increased in VLAM-treated wounds (Fig. 6a, b, right panels). The white arrows indicating αSMA- (Fig. 6a, right panel) and CD31-positive (Fig. 6b, right panel) blood vessels were representative of the formation of more blood vessels at day 14 in VLAM-treated than in control wounds. On average, we observed a fourfold increase in CD31 staining in VLAM-treated wounds versus control wounds (9.08 ± 3.58 vs. 2.12 ± 2.31; *n* = 4, *p* = 0.0171).

The dermal-epidermal interface, also known as the basement membrane, is crucial for restoring normal skin architecture.^15^ Tissue sections, post-wound closure, were visualized for integrity of basement membrane by collagen IV staining of VLAM-treated and control wound tissue (Fig. 6c). White arrowheads highlight the basement membrane in VLAM-treated tissue (Fig. 6c, right panel) that remained absent in control wounds (Fig. 6c, left panel). Altogether, these data demonstrate that VLAM treatment allows for improved neovascularization and basement membrane formation, both of which are absent in control, non-healed wounds.

## Discussion

Previously, we showed that VLAM, similar to cryopreserved AM, retains all components and properties of fresh AM. However, in contrast to fresh and cryopreserved tissue, VLAM can withstand extended storage at ambient temperature.^7^ In this study, we used a diabetic mouse (db/db) model of impaired wound healing to evaluate the molecular mechanisms of VLAM activity *in vivo*. In the well-established diabetic model, wound healing is delayed due to an increase in the levels of proinflammatory cytokines and oxidative stress in the wound microenvironment.^9,16–18^

We show that the rate of wound closure in VLAM-treated animals was faster than in both DLAM-treated and control groups (Fig. 1a). AM has been shown to support both wound granulation and re-epithelialization via modulation of fibroblast and keratinocyte proliferation and migration.^19–23^ The deposition and maturation of collagen, two steps in granulation tissue formation, are required for restoration of the dermal-epidermal junction and chronic wound re-epithelialization.^1,24,25^ VLAM treatment resulted in wound closure with a complete restoration of the dermal-epidermal junction (Figs. 2c, f, and 6c, right panel). In contrast, a continuous dermal-epidermal junction was not observed in the control after wound closure (Figs. 2a, b, d, e and 6c, left panel).

Staining for αSMA and CD31, markers of blood vessel-forming cells, and for collagen IV, a basement membrane marker, showed better neovascularization in the wound bed (Fig. 6a, b) and a fully regenerated basement membrane in VLAM-treated wounds as compared with control wounds (Fig. 6c). Collectively, these results demonstrate that VLAM promotes rapid wound closure with restoration of normal tissue architecture in both dermal and epidermal skin layers.

The normal wound-healing process involves several overlapping phases.^1^ In db/db mice, wounds fail to proceed through the normal healing process in a timely manner; wound healing is stalled in the inflammatory phase.^16,26^ After 1 week of VLAM treatment, we observed a significant shift in gene expression in cells from the wound area (Fig. 3a, b), indicative of a reduction in inflammation. These gene expression data correlate with the positive changes in observed protein levels of proinflammatory and anti-inflammatory cytokines as detected by a multiplex array at 7 and 14 days post-wounding (after one or two applications of VLAM, respectively) (Fig. 4).

Real-time PCR confirmed a marked decrease in the expression of proinflammatory cytokine *IL-1*β in VLAM-treated wounds as compared with the control (Fig. 3c). A sustained increase in IL-1β by macrophages in both human chronic wounds and db/db mouse wounds has been described in the literature. A previous study reported that the inhibition of IL-1β expression using an IL-1β neutralizing antibody led to a reduction in proinflammatory macrophage phenotype and improved wound healing in db/db mice.^27^

TNF-α, another multifunctional proinflammatory cytokine, is a predictive marker for delayed wound closure.^28,29^ Elevated levels of TNF-α in tissue and fluid collected from db/db mouse wounds and human chronic wounds have been reported in the literature.^29–31^ Inhibition of TNF-α activity using a TNF-α neutralizing antibody *in vivo* significantly enhanced wound closure in diabetic mice with an increased number of proliferating fibroblasts and migrating keratinocytes.^28,32^ Further, neutralization of TNF-α activity using soluble tumor necrosis factor receptor 1 increased diabetic wound angiogenesis *in vivo.*^33^ In this study, we detected a noticeable reduction in TNF-α levels by using both gene expression and a multiplex protein array (Figs. 3b, c and 4f). A decrease in TNF-α in VLAM-treated wounds *in vivo* correlates with a previously reported inhibition of TNF-α release from stimulated human peripheral blood mononuclear cells *in vitro* in the presence of VLAM.^7^ An increase in anti-inflammatory cytokines IL-4 and IL-10 in VLAM-treated wounds further supports the observed improvement of an anti-inflammatory wound microenvironment in db/db mice. The chronic wound milieu as published in numerous reports is linked to proinflammatory M1 macrophage polarization.^12,34,35^ It is tempting to speculate that the downregulation of IL1β and TNF-α and the upregulation of IL-4 and IL-10 by VLAM in db/db mouse wounds leads to a shift in macrophage polarization.

Another characteristic of diabetic chronic wounds is an increase in oxidative stress.^36^ The levels of ROS are controlled by antioxidants, including ROS scavenging enzymes. It has been reported that the antioxidant activity of ROS scavengers is significantly decreased in chronic wounds.^13,36,37^ A high level of ROS is associated with inflammation and the chronic nature of these wounds.^38,39^ Here, VLAM treatment resulted in an increase in the activities of catalase and glutathione peroxidase, two major ROS-scavenging enzymes (Fig. 5). Previously, it was reported that antioxidant activity of these enzymes is beneficial for healing of diabetic mouse chronic wounds *in vivo*.^9^ On VLAM treatment, we observed a positive shift in cytokine gene and protein level expression profiles from proinflammatory to anti-inflammatory as well as an increase in antioxidant enzyme activity. These observed changes in the wound microenvironment support the transition from the inflammatory phase to the proliferative phase of wound healing. The shift in the wound microenvironment from proinflammatory toward anti-inflammatory after VLAM treatment compared with the control correlates to ∼50% higher wound size reduction in VLAM-treated animals than in the control group at days 7 and 14, respectively (Fig. 1a, top and bottom panels).

Although it is known that AM has properties that are beneficial for chronic wound management, only a few studies have investigated the effects of AM on a chronic wound microenvironment *in vivo* in a diabetic wound model. In one study, Zheng *et al.* reported accelerated wound healing in db/db mice treated with a cryopreserved dermal substitute composed of a micronized acellular amnion seeded with human dermal fibroblasts.^23^ Similar observations were reported in another study conducted by Zheng *et al.* in which wounds in diabetic mice were treated with cryopreserved living micronized amnion.^40^ The authors concluded that rapid closure after application of the cryopreserved living micronized amnion to wounds in db/db mice was mediated mainly by a paracrine mechanism. Growth factors and cytokines secreted by cells in the micronized amnion regulated macrophage migration and phenotype switch, and they recruited progenitors involved in neovascularization. The activity of devitalized micronized amnion was significantly lower than that of viable tissue.^40^ Our results are consistent with those reported by Zheng *et al.* with respect to cryopreserved micronized viable and devitalized amnions.^40^ However, our study is the first to report the molecular mechanisms of VLAM activity responsible for the observed accelerated wound closure in diabetic mice. Our VLAM lyopreservation method is unique in that it can be broadly used for living tissue preservation. In contrast to traditional tissue lyophilization methods that compromise tissue viable cells during the freeze-drying process, our method preserves viable endogenous amniotic cells. The preservation of VLAM cell viability and functionality observed in this study validates our novel tissue lyopreservation process. In comparison to cryopreserved viable AM formulations, VLAM has a tremendous advantage of storage at ambient temperatures, making it accessible for use in various applications and in most environmental settings.

Key Findings

- VLAM treatment accelerates the rate of wound closure in diabetic mice whereas DLAM does not. This finding demonstrates the importance of endogenous AM viable cell preservation.
- VLAM modulates a diabetic wound environment: reduces inflammation and oxidative stress via a decrease in proinflammatory cytokines and chemokines and an increase in anti-inflammatory factors and activity of enzymes-ROS scavengers.
- VLAM supports granulation tissue formation and neovascularization, resulting in an improved quality of regenerated skin tissue.
- The lyopreservation technique described for viable amnion preservation has tremendous practicality. The method has potential to be used for the development of novel ambient stored cell and tissue therapies.

## Innovation

For the first time, the effects of VLAM in a diabetic wound microenvironment were investigated. The application of VLAM resulted in rapid wound closure in comparison to delayed closure observed in control and DLAM-treated wounds. We found that VLAM decreases inflammation and oxidative stress. These observed positive changes in the diabetic wound microenvironment correlated with better quality of regenerated tissue. This study demonstrates that monitoring of inflammatory biomarkers in chronic wounds could be used in the development of novel wound therapies. This study also demonstrates the benefit of using living amnion to correct impaired wound healing and support skin regeneration.

## About the Authors

**Sandeep Dhall, PhD,** is currently a senior scientist at Osiris Therapeutics, Inc. (OTI). He obtained his degree in Bioengineering and postdoctoral training in Cell Biology and Neuroscience from the University of California, Riverside, CA. His work focused on the mechanisms underlying chronic and burn wound healing. **Tyler Hoffman, BS,** was a research associate at OTI when this study was conducted. Currently, he is a PhD student at the Department of Bioengineering at the University of California, Los Angeles, CA. **Malathi Sathyamoorthy, PhD,** is currently the Director of Research and Development at OTI. She earned her degree in Biochemistry and Molecular Biology from the University of Maryland, Baltimore, MD. **Anne Lerch, BS,** is a senior research associate at OTI. **Vimal Jacob, BS,** is a research associate at OTI. **Matthew Moorman, BS,** was a senior research associate at OTI when this study was conducted. Currently, he is a medical student at Philadelphia College of Osteopathic Medicine, Philadelphia, PA. **Jin-Qiang Kuang, MD,** is a Principal Scientist at OTI. **Alla Danilkovitch** is Chief Scientific Officer at OTI. She earned her PhD in Cell Biology from Moscow State University, Russia. She has more than 25 years of research experience, including stem cells, immunology, and cancer biology.

## Abbreviations and Acronyms

αSMA: alpha smooth muscle actin
AM: amniotic membrane
CD: cluster of differentiation
DAPI: 4′,6-diamidino-2-phenylindole
DLAM: devitalized lyopreserved amniotic membrane
DPBS: Dulbecco's phosphate-buffered saline
G-CSF: granulocyte colony stimulating factor
GM-CSF: granulocyte-macrophage colony-stimulating factor
IL: interleukin
IL17A: interleukin 17 A
KC: keratinocyte chemoattractant
mTorr: millitorr
qPCR: quantitative PCR
RNA: ribonucleic acid
ROS: reactive oxygen species
SD: standard deviation
TNF-α: tumor necrosis factor α
VCAM: viable cryopreserved amniotic membrane
VLAM: viable lyopreserved amniotic membrane
